# High-intensity acute exercise impacts motor learning in healthy older adults

**DOI:** 10.1038/s41539-024-00220-2

**Published:** 2024-02-17

**Authors:** Eleanor M. Taylor, Claire J. Cadwallader, Dylan Curtin, Trevor T.-J. Chong, Joshua J. Hendrikse, James P. Coxon

**Affiliations:** 1 School of Psychological Sciences, Turner Institute for Brain and Mental Health, Monash University, Melbourne, VIC 3800 Australia; 2 Department of Neurology, Alfred Health, Melbourne, VIC 3004 Australia; 3 Department of Clinical Neurosciences, St Vincent’s Hospital, Melbourne, VIC 3065 Australia

**Keywords:** Consolidation, Human behaviour, Motor control, Cognitive ageing

## Abstract

Healthy aging is associated with changes in motor sequence learning, with some studies indicating decline in motor skill learning in older age. Acute cardiorespiratory exercise has emerged as a potential intervention to improve motor learning, however research in healthy older adults is limited. The current study investigated the impact of high-intensity interval exercise (HIIT) on a subsequent sequential motor learning task. Twenty-four older adults (aged 55–75 years) completed either 20-minutes of cycling, or an equivalent period of active rest before practicing a sequential force grip task. Skill learning was assessed during acquisition and at a 6-hour retention test. In contrast to expectation, exercise was associated with reduced accuracy during skill acquisition compared to rest, particularly for the oldest participants. However, improvements in motor skill were retained in the exercise condition, while a reduction in skill was observed following rest. Our findings indicate that high-intensity exercise conducted immediately prior to learning a novel motor skill may have a negative impact on motor performance during learning in older adults. We also demonstrated that exercise may facilitate early offline consolidation of a motor skill within this population, which has implications for motor rehabilitation.

## Introduction

Motor learning, the process by which motor tasks become more efficient and automatic with practice, is crucial for daily functioning. Motor learning involves both *online* acquisition of the new skill, when the skill improves during practice, and *offline* consolidation, which occurs between practice sessions. Formation of a ‘motor memory’ requires the learning trace to be encoded into memory and become resistant to interference^1^. Aging has been associated with changes in both online acquisition and offline consolidation^2,3^, which may impact the ability to acquire new skills, and reduce efficacy of motor rehabilitation^4–6^. Studies suggest the rate and magnitude of motor learning acquisition for older adults is similar to younger adults^7–11^, however, learning is diminished in tasks with increased complexity or cognitive demands^12–15^. Similarly, older adults show reduced offline consolidation compared to younger adults, as demonstrated by poorer performance on retention tests^10,16–18^ and greater susceptibility to interference^19^. One potential intervention to support motor learning is acute exercise, which has been shown to benefit young adults^20^.

Increasing evidence suggests exercise can benefit both acquisition and consolidation stages of motor learning (for review see ref. 20). While some studies show that a single bout of acute cardiorespiratory exercise can improve motor performance and online learning^21–23^ accumulating evidence indicates that exercise is particularly beneficial in enhancing the offline consolidation of a new motor skill^24–27^. Although several studies have found no apparent benefit of acute exercise on motor consolidation^28–31^, a recent meta-analysis suggests this may be explained by variations in motor learning tasks and exercise characteristics. Exercise intensity appears to be of particular importance, as greater benefits have been identified following high-intensity exercise (76–95% Maximal heart rate) compared to moderate (64–75% maximal heart rate) or light intensity exercise (57–63% maximal heart rate)^32–34^. In addition to healthy young adults, there is emerging evidence that exercise can improve motor learning in clinical populations such as Parkinson’s disease^35,36^, and Huntington’s disease^37^ however this represents a relatively new area of research, and few large-scale studies have been conducted within these populations. Similarly, research into the benefits of acute exercise on motor learning in healthy older adults is limited.

In older adults, greater cardiorespiratory fitness and increased engagement in leisure activities are associated with better motor sequence learning^38^ and greater capacity to induce plasticity in the motor cortex^39^. However, a single bout of exercise may be more accessible compared to longer exercise interventions for older adults, who face increased barriers to exercise^40^. Furthermore, the effects of acute exercise on memory are distinct from those observed in chronic exercise^41^ and the impact of acute exercise on motor learning in healthy older adults remains unclear. Hubner et al.^23^ found that older adults improved performance on a precision grip force-matching task immediately following an acute bout of moderate-intensity exercise compared to non-exercised controls, although they found no benefit of exercise on acquisition or consolidation of learning. Similarly, Greeley et al.^42^ found no effect of interval exercise on an implicit motor learning task in older adults. Notably, the motor tasks in these studies did not involve explicit sequence learning. Improvements in motor learning are proposed to relate to cognitive aspects of learning, rather than purely motoric improvements^43^, and exercise has been shown to benefit different aspects of cognition in older adults^44–46^. Importantly, while these studies found no apparent effect of exercise on behavioural measures of motor learning, they did show evidence of increased cortical activity^23^ and resting state functional connectivity^42^, respectively, in brain regions associated with motor learning. Therefore, the benefit of acute exercise in older adults may become evident at higher exercise intensities, and in motor learning tasks with greater cognitive demands.

The current study aimed to investigate whether acute high-intensity exercise can improve motor learning of a complex motor skill in healthy older adults. The sequential visual isometric pinch task (SVIPT) in the current study^26,37^ required participants to learn explicitly cued sequences and an implicit force-to-cursor movement transformation, thereby relying on both explicit and implicit learning processes^47,48^. Research utilising similar tasks identified engagement of cortical regions associated with distinct learning components including declarative sequence learning and sensorimotor mapping^49^. The added complexity of this continuous force modulation task may be more comparable to real-life skills compared to discrete motor sequence tasks such as finger-tapping^43^. It was hypothesised that healthy older adults who completed a bout of high-intensity exercise would demonstrate improved acquisition and offline consolidation of a novel motor skill compared to those who completed an equivalent period of rest. Chronological age effects across the older adult sample were also investigated, as ageing is a continuous process and changes in physical and cognitive function are common across the age ranges included in the study.

## Results

**Fig. 1:**
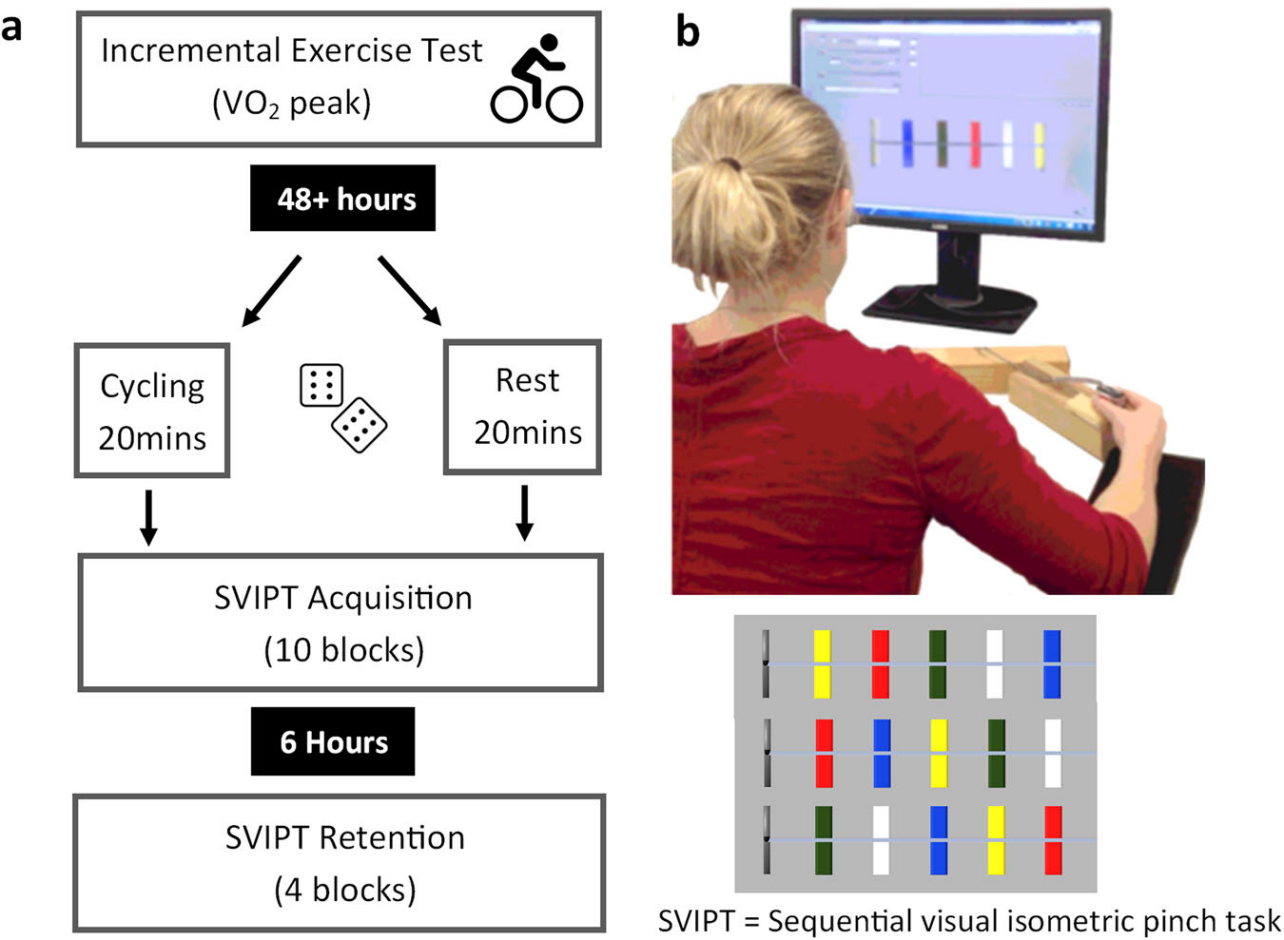
Overview of the study design. **a** Overview of testing schedule. An incremental exercise test was conducted at least 48 hours before subsequent testing. Participants were randomised into Rest or Exercise conditions. Acquisition and retention of the motor task were completed on the same day with a 6 ± 1-hour delay between testing. **b** Depiction of SVIPT motor task adapted from Stavrinos & Coxon^26^. In this version of the SVIPT, three motor sequences are presented in a pseudorandom order within each block of 12 trials. This is a more cognitively challenging version of the SVIPT that requires the trial-to-trial recall, planning, execution, and learning of multiple sequences.

Participants were 24 right-handed healthy adults aged 55−75 years (*M*_age_ = 66.68 years, SD = 5.32). The study utilised a between-groups design, with participants randomly allocated to either an exercise or active rest (control) group. The experimental session involving a 20-minute bout of high-intensity interval exercise (HIIT) or an equivalent period of active rest. Following exercise or rest, participants completed a novel computer-based task, a variant of the sequential visual isometric pinch task (SVIPT)^26^ to assess motor skill acquisition (Fig. 1a). Retention of the novel motor skill was assessed on the same day following a 6-hour break during which participants were asked to refrain from exercise or sleep. Acquisition and retention data were assessed using linear mixed model analyses. Please see the Methods section and supplementary materials for a complete summary of variable selection and model fitting.

### Baseline

Group characteristics are summarised in Table 1. Groups were balanced on age, sex, body mass index, physical activity level, resting heart rate, and retention test delay (all *p* > 0.05). Baseline skill scores (block 1) did not differ across Group (*t*(21) = 0.13, *p* = 0.89), nor did force error (*t*(21) = −0.22, *p* = 0.83) or trial time (*t*(21) = −0.05, *p* = 0.97).

**Table 1:** Participant means and standard deviations for Rest and Exercise groups

	Rest	HIIT exercise
*n* (female)	12 (7)	11 (8)
Age (years)	67.92 ± 4.10	65.27 ± 6.31
Retention test delay (hrs)	5.89 ± 0.36	5.77 ± 0.30
Body mass index	25.56 ± 3.59	27.19 ± 4.54
Self-report physical activity (IPAQ)	5789 ± 3888	8224 ± 3725
Maximal fitness test		
VO_2_peak (mL.kg^−1^.min^−1^)	36.68 ± 10.92	38.76 ± 11.31
*n* maximal test attained	4	7
Resting HR	64.25 ± 3.41	67.00 ± 11.51
Max HR (attained)	151.00 ± 14.85	158.45 ± 11.56
Max HR (estimated)	160.46 ± 2.87	162.31 ± 4.42
Max output (W)	187.27 ± 71.65	185.00 ± 97.25
Exercise session characteristics		
Peak HR	82.82 ± 10.40	150.91 ± 13.64
Peak output (W)		150.03 ± 83.85
Peak %HRR		91.09 ± 10.46
RPE		16 ± 2.79

Resting heart rate (HR), max HR measured in beats per minute. Max output in watts (W), and heart rate reserve (HRR) obtained from graded exercise test. IPAQ (International Physical Activity Questionnaire) scores expressed as METs minutes/week. There were no significant group differences in demographics or maximal fitness parameters (all *p* > 0.05). Peak HR, output and Borg’s Rating of Perceived Exertion (RPE) for the acute exercise bout summarised for the exercise group only.

### Acquisition

**Fig. 2:**
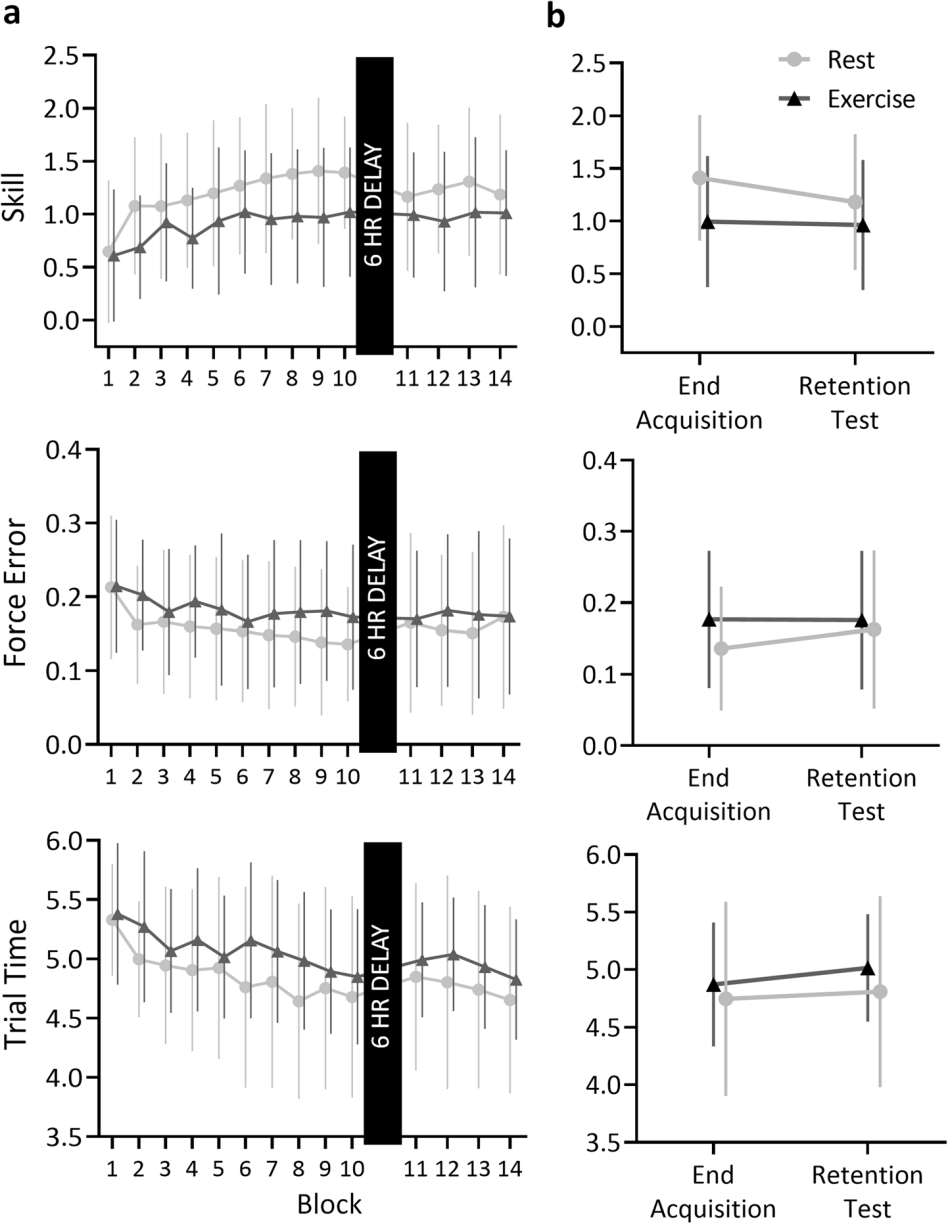
Summary of SVIPT performance by group. Data are presented as observed means ± standard deviation. Lower force error and trial time reflect higher skill. **a** Mean scores for skill, force error and trial time by group. **b** Change in scores between end of learning and retention test. For the rest group there was a reduction in skill and an increase in force error following the 6-hour delay. The exercise group were not susceptible to these detriments in skill and force error. Scores did not differ between groups at either time point.

**Fig. 3:**
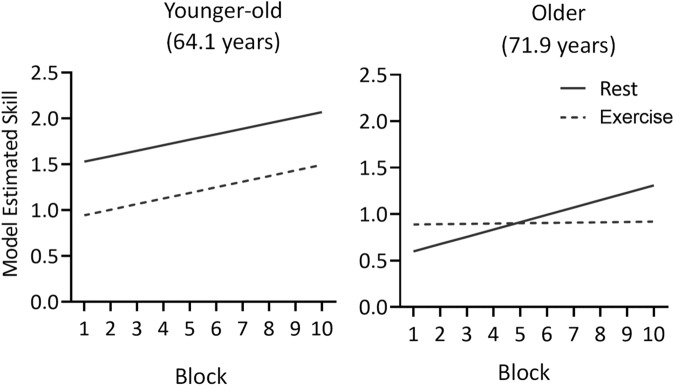
Model estimates demonstrating the impact of exercise on skill across different ages. The change in skill over time was assessed at one standard deviation above and below the mean age. Older age (71.9 years) was associated with an increase in skill across blocks for the rest group but not exercise. Younger age (64.1 years) was associated with increased skill across blocks for both exercise and rest groups.

SVIPT performance across all blocks is summarised in Fig. 2a. For skill scores, the linear mixed model analysis (Table 2) revealed several main effects, including Group (*F*(1, 17.00) = 5.71, *p* = 0.03) with exercise participants scoring a total of 6.58 lower than rest across all blocks (95% CI [−12.01, −1.15]), and Age (*F*(1, 17.00) = 5.74, *p* = 0.03) as younger-old participants scored 0.71 higher than older participants (95% CI [0.13, 1.29]). However, these main effects were superseded by the interaction effects in the model. A full summary of main effects, interactions and fixed effects estimates for all linear mixed models can be found in the Supplementary Materials, but most notably, there was a significant three-way interaction between Group, Block, and Age (*F*(1, 19.00) = 4.61, *p* = 0.04). Figure 3 shows model estimates for change in skill across Block for the rest and exercise groups at different ages. Within this older adult sample, younger-old age was associated with significant improvement across both rest ($\hat{\beta }$ = 0.06, 95% CI [0.01, 0.11], *p* = 0.03) and exercise groups ($\hat{\beta }$ = 0.06, 95% CI [0.03, 0.09], *p* = 0.001). In contrast, older age was associated with improvement in skill across Block in the rest group ($\hat{\beta }$ = 0.08, 95% CI [0.03, 0.12], *p* = 0.001), but not following exercise ($\hat{\beta }$ = −0.001, 95% CI [−0.04, 0.04], *p* > 0.99).

**Table 2:** Summary of linear mixed models

Dependent variable	Model predictors	AIC	*R*^2^(m)	*R*^2^(c)
Acquisition				
Skill	~ Group × block × age + sex + fitness + (block | participant)	106.15	0.39	0.89
Force error	~ Group × block × age + sex + fitness + (1| participant)	−778.72	0.32	0.87
Trial time	~ Group × block × age + (block | participant)	141.66	19	0.89
Retention				
Skill	~ Group × time + age + sex + fitness +(1 | participant)	37.21	0.33	0.96
Force error	~ Group × time + age + sex + fitness +(1 | participant)	−130.99	0.25	0.96
Trial time	~ Group × time + (1 | participant)	63.37	0.02	0.91

*AIC* Akaike Information Criterion. Smaller AIC values reflect better model fit. AIC values are derived from model fit using restricted maximum likelihood (REML). *R*^2^(m) marginal *R* squared. A larger *R*^2^(m) reflects a higher proportion of variance accounted by fixed factors alone. *R*^2^(c) Conditional *R* squared. A larger *R*^2^(c) indicates a higher proportion of variance explained by both fixed and random factors.

Differences in skill were driven by changes in the accuracy subcomponent of the skill measure. Force error scores showed a three-way interaction between Group, Block and Age (*F*(1, 195.00) = 5.70, *p* = 0.02). Younger-old participants reduced force error following rest ($\hat{\beta }$ = −0.01, 95% CI [−0.01, −0.001], *p* = 0.02) and exercise ($\hat{\beta }$ = −0.01, 95% CI [−0.01, −0.003], *p* <.001), while older participants reduced error following rest ($\hat{\beta }$ = −0.01, 95% CI [−0.01, −0.002], *p* = 0.004), but not exercise ($\hat{\beta }$ = 0.002, 95% CI [−0.002, 0.01], *p* = 0.45).

Analysis of trial time revealed no significant main effect of Group (*F*(1, 19.00) = 0.72, *p* = 0.40), though there was a main effect of Block (*F*(1, 19.00) = 6.29, *p* = 0.02, $\hat{\beta }$ = 0.83, 95% CI [0.15, 1.51]) and a Block by Age interaction (*F*(1, 19.00) = 7.62, *p* = 0.01). Model estimates indicate that older participants decreased speed over time ($\hat{\beta }=$−0.09, 95% CI [−0.14, −0.04]) while younger-old did not ($\hat{\beta }$ = −0.002, 95% CI [−0.05, 0.05]), however this did not vary across rest and exercise (Group × Block × Age: *F*(1, 19.00) = 2.18, *p* = 0.16).

### Retention

Assessment of skill retention, summarised in Fig. 2b, revealed no main effect of Group (*F*(1, 18) = 1.79, *p* = 0.20), however there was a Group by Time interaction (*F*(1, 21) = 5.98, *p* = 0.03), with participants in the rest group showing a reduction in skill between learning and retention ($\hat{\beta }=$−0.09, 95% CI [−0.34, −0.11]) while participants in the exercise group showed no change in skill over the delay ($\hat{\beta }=$−0.03, 95% CI [−0.15, 0.55]). However, skill scores did not differ significantly between exercise and rest groups at the end of acquisition ($\hat{\beta }=$0.19, 95% CI [−0.32, 0.70]) or the start of the retention test ($\hat{\beta }=$−0.22, 95% CI [0.73, 0.29]).

Assessing the force error subcomponent of skill, there was no effect of Group (*F*(1, 18) = 0.45, *p* = 0.21). However, a Group by Time interaction (*F*(1,2 1) = 4.50, *p* = 0.05) revealed that force error increased at the retention test for the rest group ($\hat{\beta }=$0.03, 95% CI [0.01, 0.04]) but not for the exercise group ($\hat{\beta }=$−0.001, 95% CI [−0.02, 0.02]).

## Discussion

The current study aimed to investigate the effect of acute high-intensity exercise on motor learning in healthy older adults. The current study had two main findings. First, high-intensity exercise did not benefit acquisition of motor learning in healthy older adults. Contrary to expectation, exercise did not facilitate motor learning acquisition, and for some participants exercise had a negative impact. Second, the reduction in skill observed in the rest group between the end of acquisition and the retention test was eliminated in the exercise group, suggesting that HIIT exercise improved consolidation of the motor skill. This resulted in equivalent performance across groups at the retention test, despite the relatively poorer performance during acquisition following exercise. As discussed below, these promising findings can inform the application of acute high-intensity exercise to support motor learning in older populations.

Consistent with extant literature regarding young adults^20^, the current study found no benefit of acute exercise on online learning of a motor sequence learning task in healthy older adults. Indeed, exercise had a detrimental impact on motor performance during acquisition, though this was specific to the oldest participants in our study. This contrasts with findings from Hubner and colleagues^23^ who reported improved motor performance in healthy older adults following a moderate-intensity exercise intervention. The differing results in the current study may relate to the use of high-intensity exercise, and a more cognitively complex motor task. The current findings also contrast with preliminary findings in clinical populations such as Parkinson’s disease, wherein exercise was found to have no impact on motor acquisition during serial reaction time^36^ and whole-body balance tasks^35,36^. Notably these studies also utilised moderate intensity exercise protocols, rather than high-intensity as in the current study. The current findings provide preliminary evidence that exercise has a greater negative impact on motor performance with increasing age, however this warrants further investigation within a larger sample.

Although increased physiological arousal associated with exercise has been shown to enhance basic cognitive process such as attention, rapid decision making, and automatic responses^50,51^, the impact on higher-order, complex cognitive processes (i.e., those required for online acquisition of our modified SVIPT task) is less clear. The relationship between arousal and complex cognitive functions is thought to be U-shaped^52–54^ and higher intensity exercise may be detrimental, at least to some types of cognition^55^. In addition to arousal-related deficits, fatigue may also have impacted performance during acquisition following exercise. Although fatigue-related effects of exercise on cognition are commonly identified following prolonged exercise^56^, the high intensity of exercise in the current study may have resulted in fatigue-related effects on motor performance during learning^57^.

The SVIPT task used in the present study is complex, involving both implicit motor adaptation learning and explicit sequence learning components. The findings of the current study show that high-intensity exercise may be detrimental to performance of cognitively complex motor learning tasks by healthy older adults. This effect varied according to age within our sample, supporting the importance of optimising acute exercise interventions based on individual characteristics.

The current study found no overall difference in motor skill between groups at the start of the retention test. This indicates that learning of the motor sequences occurred for both groups, suggesting that high-intensity exercise may have negatively impacted motor *performance* for older participants, but not necessarily *learning*. Older age is associated with reductions in online motor learning relative to younger adults^58–60^ which may reflect age-related changes in cortical and subcortical activity^61^. For example, several studies suggest older adults show a differential pattern of cortical activation in response to motor demands^62–65^ and following motor learning^66^ relative to young adults. Furthermore, King and colleagues^15^ found that changes in functional connectivity in older adults, e.g. reduced segregation of large-scale resting networks, are associated with poorer motor performance, particularly in cognitively complex tasks. Notably, there is some evidence that high-intensity exercise may have a differential impact on the activity of these networks during attentionally-demanding tasks, compared to moderate-intensity or no exercise^67^. One interpretation of the current results is that older participants were more susceptible to arousal-related deficits following exercise, which impacted their performance during motor skill acquisition.

The reported age-related deficits in motor learning in the literature likely reflect changes in offline consolidation, as older adults tend to show reduced offline improvements following motor learning compared to younger adults^2,18^, and are more sensitive to interference^19^. In young adults, exercise is most frequently reported to benefit consolidation of motor learning, rather than online acquisition, particularly following high-intensity exercise^34^. Importantly, the current results suggest that high-intensity exercise also benefits motor learning consolidation in older populations.

At the retention test, a reduction in performance was observed in the rest group, but not the exercise group, who showed no significant change in skill following a delay. This is evidence that exercise supported early consolidation processes, allowing for greater stabilisation of the newly learned motor skill. Although skill was retained ~6 hours following exercise, this is in contrast to previous findings using a similar experimental protocol in younger adults, which found an improvement in skill relative to a rested control group at the retention test^26^. This likely reflects changes in consolidation associated with aging. While some motor learning tasks are associated with offline stabilisation or improvement in younger adults, this is often reduced in older participants^3,17^, and can be replaced by offline forgetting^68^. When contrasted with studies using similar learning tasks^26^, the current findings suggest that high-intensity exercise may benefit early consolidation processes relative to rest in older adults by reducing offline forgetting, rather than facilitating offline improvement.

The current findings contrast with those of Greeley and colleagues^42^, who found no benefit of exercise on implicit motor sequence learning in older adults. Key methodological differences between the Greely et al. study^42^ and the current study may explain these divergent findings. Most notably, the current study utilised a higher intensity exercise protocol, with participants reaching up to 90% of their estimated maximum heart rate during high-intensity epochs, resulting in greater power output and overall exertion compared to Greeley and colleagues^42^. Although high-intensity exercise may impact motor skill acquisition, there appears to be a dose-response relationship in favour of higher-intensity exercise in supporting motor consolidation^33,34^. This may relate to the cascade of neurochemical changes induced by high-intensity exercise, which may maintain an interval environment favourable for memory consolidation, including increased circulation of catecholamines^69^, lactate^70^, and brain-derived neurotrophic factor (BDNF)^71^. Notably, studies involving participants with Parkinson’s disease have shown motor learning improvements following moderate intensity exercise^35,36^. Individuals with age-related neurodegenerative disorders may demonstrate benefits from exercise on motor learning at a lower intensity compared to healthy older adults, due to the impact of exercise on disease related processes^72^.

The benefits of acute exercise on motor learning are dependent on the characteristics and timing of the exercise and motor learning task. Greater improvements in new skills have been observed when exercise is completed in close temporal proximity to motor learning (e.g., immediately before or after learning^73^). Interestingly, participants who complete exercise after a motor learning task have demonstrated greater retention of the skill compared to those who exercise prior to learning^74^. Wanner et al.^20^ suggest that exercise prior to learning may improve acquisition and early consolidation of the skill, while exercise following learning improves the ongoing consolidation of the skill. The present study suggests this is not the case in healthy older adults, as acute exercise prior to motor learning conferred no apparent benefit on learning acquisition.

There are likely complex interactions between the timing of exercise, and the characteristics of the exercise, learning task, and individual. Stavrinos and Coxon^26^ found no negative effect of acute high intensity exercise on motor performance in healthy young adults, despite using a similar study design and task to the current research. Similarly, Hubner et al.^23^ showed that moderate intensity exercise prior to learning in older adults is beneficial for basic motor performance, however it is unclear whether this holds true in cognitively complex motor learning tasks such as that used in the current study. Further research is required to investigate the interplay between exercise and task characteristics in healthy older adults to better identify the optimal application of exercise in this population. In particular, identifying the appropriate timing and intensity of exercise for this age group to maximise the benefit on motor consolidation, while minimising the impact on motor performance.

The current study employed a moderate sample size, determined by power analysis based on previous studies using comparable motor learning tasks^26^. We note that while the groups were matched overall, the demographic variables of age and cardiorespiratory fitness were found to be relevant to the study outcomes. These warrant further investigation, as age-related changes in motor learning may not occur in a linear fashion across the age range included in the current study^58^. Additional studies with larger samples are required to robustly examine interactions between these demographic variables on observed outcomes.

This study also utilised a same-day retention test, minimising potential confounding factors of sleep quality on subsequent motor skill consolidation and providing novel insight into the benefit of exercise on early consolidation. While sleep is not necessary for early motor consolidation^26^, we note that sleep-dependent consolidation is a key aspect of motor learning^75,76^ and changes in sleep architecture may play a role in age-related declines in motor learning^77,78^. Moreover, previous exercise and motor learning studies suggest the benefits of exercise on consolidation become more pronounced over days and weeks after learning in younger adults^20^. It is unclear whether sleep dependent consolidation would enhance exercise-related benefits in an older population, or whether benefits would be limited by age-related changes in sleep architecture. Further research is required to examine the longer-term impact of HIIT exercise on complex motor learning in older adults.

Comparison of motor retention is complicated by the effect of exercise on motor performance during acquisition. For example, it is possible that differences noted at the retention test relate to ceiling effects in the rest group. Notably, previous studies have demonstrated improvement in SVIPT tasks across training multiple sessions and over multiple days^79,80^. It is therefore unlikely that the rest group reached ceiling performance during a single training session of the task. Nevertheless, the potential impact of exercise on rate and level of skill acquisition is an important consideration in interpreting the current results.

For our motor skill learning task, exercise did not facilitate online acquisition and may even have inhibited online acquisition for the oldest participants. However, we observed preserved motor learning following a delayed retention test for participants who exercised, in contrast to participants who did not exercise that demonstrated a performance decrement. Overall, these results demonstrate the importance of individual factors such as age when designing exercise interventions. Furthermore, these results suggest that the benefits of high-intensity exercise on early motor consolidation extend to older adult populations. These findings have implications for supporting older adults in motor rehabilitation settings, providing a potential avenue to ameliorate reductions in motor learning associated with age.

## Methods

### Participants

Participants were recruited from the Melbourne area via flyers and outreach to local community groups. All participants took part in the study voluntarily and provided written consent prior to participation. Exclusion criteria included a history of neurological conditions, contraindications to exercise (Adult Pre-Exercise Screening System^81^), and cognitive impairment (as determined by a score of <26 on the Montreal Cognitive Assessment^82^). One participant was excluded from analysis due to technical issues during data collection resulting in incomplete data. Participants were all right-handed, assessed using the Edinburgh Handedness Inventory^83^. The study was approved by the Monash University Human Research Ethics Committee.

A target sample size of 28 participants was determined based on an effect size of *d* = 0.96 as reported in Stavrinos & Coxon^26^ for a Time × Exercise Condition interaction to assess motor learning retention (alpha = 0.05, power = 80%).

### Design

The study utilised a between-groups design, with participants randomly allocated to either an exercise or active rest (control) group. All participants attended an initial session wherein their cardiorespiratory fitness level was determined by measuring peak oxygen consumption (VO_2_peak) during a graded exercise protocol. After a delay of at least 48 hours, participants then returned for an experimental session involving a 20-minute bout of high-intensity interval exercise or an equivalent period of active rest. Following exercise or rest, participants completed a novel computer-based task, a variant of the sequential visual isometric pinch task (SVIPT)^26^ to assess motor skill acquisition (Fig. 1a). Retention of the novel motor skill was assessed on the same day following a 6-hour break during which participants were asked to refrain from exercise or sleep. Participants were informed that the study aimed to investigate exercise and learning. Participants were blinded to key aspects of the study including the existence of exercise and active rest groups and the expected outcomes of the study until after their participation concluded.

### Cardiorespiratory fitness test

Cardiorespiratory fitness was assessed via evaluation of peak oxygen consumption (VO_2_peak) during an incremental intensity exercise test on a stationary bicycle. All tests were supervised by an experienced exercise scientist and were conducted at least 48 hours prior to the experimental session. Prior to the test, heart rate reserve (HRR) was estimated for each participant based on age, and resting heart rate (RHR) using Eq. (1):

1 $${\rm{HRR}}={{\rm{HR}}}_{{\rm{age}}-{\rm{predicted}}\max }-{\rm{RHR}}$$

Age-predicted maximum heart rate was calculated using Eq. (2)^84^:

2 $${{\rm{HR}}}_{{\rm{age}}-{\rm{predicted}}\max }=208-(0.7\times {\rm{age}})$$

The test was preceded by a 5-minute warm up period, during which participants self-selected a pedalling cadence which was maintained for the duration of the test. The first two stages lasted 3 minutes each, with workload adjusted such that participants reached ~40% and 60% of their estimated HRR, respectively. This was followed by 1-minute stages wherein the workload was then increased by 10-15 Watts per stage. The test lasted between 8–12-minutes, terminating once participants were no longer able to maintain the self-selected cadence. Heart rate was monitored throughout the test using a Polar H10 heart rate monitor (Polar Electro, Finland). Expired air volume, and oxygen and carbon dioxide concentration were recorded using a Powerlab 16/35 and LabChart 7 data acquisition system (ADInstruments, Dunedin, New Zealand) configured to provide breath-by-breath analysis. Subjective exertion was evaluated each minute using Borg’s Rating of Perceived Exertion (RPE)^85^.

VO_2_peak was defined as the maximum oxygen consumption rate identifiable in a 20-second averaged epoch. The incremental exercise was considered a maximal test if at least two of the following indicators were met: (1) respiratory exchange ratio of ≥ 1.1, (2) heart rate ≥95% estimated HRR, or (3) a self-reported RPE of ≥ 17 out of 20^86^. For twelve participants who did not meet these criteria, individual regression equations were calculated (Mean *R*^2^ = 0.91) based on submaximal data (VO_2_ measured at 40% and 60% of estimated HRR^87^). These regression equations were used to derive a predicted VO_2_peak value based on their estimated maximum heart rate^88^.

### Exercise interventions

Both exercise and active rest protocols were completed on a Wattbike Atom stationary bicycle (Wattbike, 2017). Participant heart rate was monitored throughout using a Polar H10 heart rate monitor. Exercise intensity was calculated for each participant based on their incremental exercise test as a percentage of HRR. Participants in the exercise condition completed a 20-minute high-intensity interval (HIIT) protocol. The protocol alternated between 3-minute phases of low-intensity cycling (approximately 50%HRR) and 2-minute phases of moderate- to high-intensity cycling (up to 90%HRR)^89,90^. This was followed by a low-intensity cool-down period. Participants in the rest group completed 20 minutes of slow pedalling on the stationary bicycle such that their heart rate remained within 15 beats per minute of heart rate measured at the commencement of the session. This “active rest” aimed to have rest participants engage in the movement of the exercise bout, however with minimal to no physical exertion. All participants then completed a short, seated break (5–10 minutes) before commencing the motor learning task.

### Motor learning task

After the exercise or active rest protocol, participants completed the SVIPT task (Fig. 1b). Participants were seated before a computer holding a force transducer between their thumb and index finger of their dominant hand. Squeezing the force transducer produced a proportional on-screen cursor movement. Each trial commenced when 5 coloured targets appeared on screen. Participants were instructed to produce five pulses of force to move the cursor to the targets as quickly and accurately as possible according to a specified colour sequence (red-blue-green-yellow-white). Target locations were pseudorandomly shuffled among 3 different orders, requiring the learning and preparation of 3 different motor execution sequences. The amount of force required to reach the furthest target was set at 45% of each participant’s maximum voluntary pinch contraction (MVC).

Participants completed 9 initial trials to allow familiarisation with the task followed by 10 blocks of 12 trials, constituting the motor learning acquisition phase. To assess progress and encourage continual improvement, participants were shown a visual representation of their calculated skill level after completion of each block. Following a delay of 6 ± 1 hours (Rest *M* = 5.89 ± 0.36 hours, Exercise *M* = 5.77 ± 0.30 hours) participants returned to complete a retention test, comprising a warm-up (6 trials of the task to counteract the established warm-up decrement for this task^91^) followed by four blocks of 12 trials.

### Data analysis

Performance on the SVIPT was assessed by calculating a skill measure, with higher values reflecting a shift in the speed-accuracy trade-off function towards faster and more accurate task performance^26,79^. On each trial, accuracy was recorded as the summed distance from each of the five force peaks to their respective targets, resulting in a force error score, with lower force error indicating greater accuracy. The speed of each trial was calculated as the duration from trial onset to the end of the final force peak. The speed-accuracy trade-off function for the SVIPT has previously been defined according to Eq. (3):

3 $${\rm{Skill}}\; {\rm{parameter}}=(1-{\rm{force}}\; {\rm{error}})/({\rm{force}}\; {\rm{error}}\times (\log ({\rm{duration}})^{\rm{a}}))$$

where duration refers to the mean trial time for the block, and the value of *a* is 1.627^26^. The same formula was applied to capture the speed-accuracy trade-off function, and to ensure homogeneity of variance across participants, the logarithm of this skill parameter was taken as the skill measure used for all analyses^79^.

Data was assessed on a trial-by-trial basis. A trial was considered valid if it included 5 distinct force pulses within the timespan of the trial. Of the total 3864 trials, 267 (6.9%) were ineligible and excluded, and an additional 42 (1.1%) were identified as outliers (*z* = > ± 3.29) and Winsorized to eliminate bias prior to calculating mean performance across blocks for each participant.

### Statistical analysis

Baseline performance was assessed using independent samples t-tests. Mean skill, force error, and trial time for block 1 was compared between exercise and rest groups. To evaluate differences in skill, force error, and trial time across the 10 blocks of learning acquisition between exercise and rest groups, linear mixed models (LMMs) were constructed using a model selection approach. A maximal model-based approach was considered inappropriate for the current study, given a sample size and risk of overfitting^92^. The selected models included Participant as a random factor, random slopes for Block, and fixed effects of Group (rest, exercise) and Block (1–10). Additional variables of theoretical interest (Sex, Age, and Cardiorespiratory Fitness) and interactions were entered into the model and were retained if they significantly improved overall model fit as indicated by the Akaike Information Criterion (AIC)^93^. In cases where Chi-square comparisons between models were non-significant, the more parsimonious model was selected. For a detailed summary of model fitting, see Supplementary Material.

Retention of motor learning was assessed as the difference in performance at the end of acquisition (average across blocks 9 and 10) and the beginning of the retention test (average across blocks 11 and 12). LMMs were constructed for skill, trial time, and force error, with Participant included as a random effect and Time (end of learning, start of retention) as a fixed effect. Additional fixed effects and interactions were added according to the model fitting process described above. Significant effects were followed-up using Bonferroni-adjusted pairwise comparisons of model estimates. For ease of interpretation, significant relationships involving age were followed up by comparing scores at one standard deviation above and below the mean age (61.3 years, referred to as “younger-old”, and 71.9 years, referred to as “older”)^94^.

Models were fit in RStudio (version 2022.07.2) (RStudio Team, 2022) using the lme4 package^95^. Model comparisons were conducted with maximum likelihood estimation, with restricted maximum likelihood estimation and a Satterthwaite adjustment to compute the degrees of freedom in the final models. Selected models are described in Table 2. Overall effects, interactions and *p* values were calculated using the lmerTest package^96^. Effect sizes are represented by beta estimates and 95% confidence intervals. Results are reported as mean ± standard deviation, or model estimates and 95% confidence intervals. *α* was set to .05 for all analyses.

### Reporting summary

Further information on research design is available in the Nature Research Reporting Summary linked to this article.

### Supplementary information

Supplementary material

Reporting summary

## Data Availability

Behavioural data are available upon request by contacting the corresponding author (i.e., J.P.C).

## Code availability

Custom-written code is available upon request by contacting the corresponding author (i.e., J.P.C).
