# Supplementary material for: High-intensity acute exercise impacts motor learning in healthy older adults
Source: NPJ Sci Learn. 2024 Feb 17;9:9. doi: 10.1038/s41539-024-00220-2 (PMC10874400; doi:10.1038/s41539-024-00220-2)
Supplement: Supplementary file 1 — Supplementary material [file 41539_2024_220_MOESM1_ESM.pdf]

Supplementary Material: "High-intensity acute exercise impacts motor learning in healthy older adults"

Eleanor M. Taylor[1], Claire J. Cadwallader[1], Dylan Curtin[1], Trevor T.J. Chong[1,2,3], Joshua J. Hendrikse[1], and James P. Coxon[1]

Contents

Supplementary Methods (pp. 2 – 5)

Linear mixed model fitting

Supplementary Table 1: Model fitting for acquisition - skill measure

Supplementary Table 2: Model fitting for acquisition - force error

Supplementary Table 3: Model fitting for acquisition - trial time

Supplementary Table 4: Model fitting for retention - skill measure

Supplementary Table 5: Model fitting for retention - force error

Supplementary Table 6: Model fitting for retention – trial time

Supplementary Results (pp. 6 – 7)

Supplementary Table 7: Results of linear mixed models for acquisition

Supplementary Table 8: Results of linear mixed models for retention

Supplementary Methods

Linear Mixed Model Fitting

Supplementary tables 1-6 include a summary of model comparisons. For skill measure, force error and trial time models, variables and interactions were added based on theoretical interest and retained where model fit was improved. Model comparisons were conducted with maximum likelihood estimation. In cases where Chi-square comparisons between models were non-significant, the more parsimonious model was selected. A summary of power analysis of selected models available upon request.

Supplementary table 1. *Model fitting for acquisition - skill measure.*

		N total obs = 230; N Subjects = 23; N blocks = 10			**Model Fit**			**LRT Test against comparison**	
**Model Specification**	**Model Name**	**Compared Model**	**Model description**	**Random Effects**	**AIC**	**BIC**	**LL**	χ^2	*p*
RE only	Null		Skill ~ (1 | Participant)	Participant	197.85	208.16	-95.93		
	Skill 1	Null	Skill ~ (1|Block) + (1| Participant)	Participant, Block	141.15	154.90	-66.57	58.70	<.001*
	Skill 2	Skill 1	Skill ~ (Block | Participant)	Participant, Block	137.81	155.00	-63.91	5.34	.02*
	Skill 3	Skill 2	Skill ~ (Block | Participant) + (1 | Age)	Participant, Block, Age	139.81	160.44	-63.91	<.001	>.99
	Skill 4	Skill 2	Skill ~ (Block | Participant) + (1 | Fitness)	Participant, Block, Fitness	Model failed to converge				
FE main effects	Skill 4	Skill 2	Skill ~ Group + Block + Age + (Block | Participant)	Participant, Block	119.65	147.16	-51.83	24.16	<.001*
	Skill 5	Skill 4	Skill ~ Group + Block + Age + Sex + Fitness (Block | Participant)	Participant, Block	115.08	149.46	-47.54	8.57	.01*
FE Interactions	Skill 6		Skill ~ Group × Block + Age + Sex + Fitness + (Block | Participant)	Participant, Block	114.50	152.32	-46.25		
	**Skill 7**	**Skill 6**	**Skill ~ Group × Block × Age + Sex + Fitness + (Block | Participant)**	**Participant, Block**	**106.15**	**154.28**	**-39.08**	**14.35**	**.002***
	Skill 8	Skill 7	Skill ~ Group × Block × Sex + Age + Fitness + (Block | Participant)	Participant, Block	117.97	166.11	-44.99	2.52	0.47
	Skill 9	Skill 7	Skill ~ Group × Block × Fitness + Sex + Age + (Block | Participant)	Participant, Block	Model failed to converge				
	Skill 10	Skill 7	Skill ~ Group × Block × Age × Sex + Fitness + (Block | Participant)	Participant, Block	111.19	183.39	-34.60	8.96	.26
	Skill 11	Skill 7	Skill ~ Group × Block × Age × Fitness + Sex + (Block | Participant)	Participant, Block	111.09	183.29	-34.55	9.06	.25

Final selected model in bold.
**p* <0.5

Supplementary table 2. *Model fitting for acquisition – force error.*

		N total obs = 230; N Subjects = 23; N blocks = 10			Model Fit			LRT Test against comparison	
**Model Specification**	**Model Name**	**Compared Model**	**Model description**	**Random Effects**	**AIC**	**BIC**	**LL**	χ^2	*p*
RE only	Null		Force Error ~ (1|Participant)	Participant	-736.94	-726.62	371.47		
	Error 1	Null	Force Error ~ (1|Block) + (1|Participant)	Participant, Block	-754.52	-740.77	381.26	19.59	<.001*
	Error 2	Error 1	Force Error ~ (Block | Participant)	Participant, Block,	-771.30	-754.11	390.65	18.78	<.001*
	Error 3	Error 2	Force Error ~ (Block | Participant) + (1|Age)	Participant, Block, Age	-769.30	-748.67	390.65	.002	.96
	Error 4	Error 2	Force Error ~ (Block | Participant) + (1|Fitness)	Participant, Block, Fitness	Model failed to converge				
FE main effects	Error 5	Error 2	Force Error ~ Group + Block + Age + (Block | Participant)	Participant, Block	-776.82	-749.32	396.41	11.53	.009*
	Error 6	Error 5	Force Error ~ Group + Block + Age + Sex + Fitness + (Block | Participant)	Participant, Block	-780.84	-746.45	400.42	8.01	.02*
FE Interactions	Error 7		Force Error ~ Group × Block + Age + Fitness + Sex + (Block | Participant)	Participant, Block	-776.40	-742.02	398.20		
	Error 8	Error 7	Force Error ~ Group × Block × Age + Sex + Fitness + (Block | Participant)	Participant, Block	Model failed to converge				
	**Error 9**	**Error 7**	**Force Error ~ Group × Block × Age + Sex + Fitness + (1|Block) + (1|Participant)**	**Participant**	**-778.72**	**-734.03**	**402.36**	**8.32**	**.04***

Final selected model in bold. **p* <0.5

Supplementary table 3. *Model fitting for acquisition – trial time*

		N total obs = 230; N Subjects = 23; N blocks = 10			Model Fit			LRT Test against comparison	
**Model Specification**	**Model Name**	**Compared Model**	**Model description**	**Random Effects**	**AIC**	**BIC**	**LL**	χ^2	*p*
RE only	Null		Trial Time ~ (1 | Participant)	Participant	262.49	272.81	-128.25		
	Time 1	Null	Trial Time ~ (1 | Block) +(1 | Participant)	Participant, Block	235.53	249.29	-113.77	28.96	<.001*
	Time 2	Time 1	Trial Time ~ (Block | Participant)	Participant, Block	151.08	168.27	-70.54	86.45	<.001*
	Time 3	Time 2	Trial Time ~ (Block | Participant) + (1 | Age)	Participant, Block, Age	153.08	173.71	-70.54	<.001	>.99
	Time 4	Time 2	Trial Time ~ (Block | Participant) + (1 | Fitness)	Participant, Block, Fitness	153.08	173.71	-70.54	<.001	>.99
FE main effects	Time 5	Time 2	Trial Time ~ Group + Block + Age + (Block | Participant)	Participant, Block	143.30	170.80	-63.65	13.78	.003*
	Time 4	Time 3	Trial Time ~ Group + Block + Age + Sex + Fitness + (Block | Participant)	Participant, Block	146.25	180.63	-63.12	1.05	.59
FE Interactions	Time 5		Trial Time ~ Group × Block + Age + (Block | Participant)	Participant, Block	146.25	180.63	-63.12		
	**Time 6**	**Time 5**	**Trial Time ~ Group × Block × Age (Block | Participant)**	**Participant, Block**	**141.66**	**182.91**	**-58.83**	**9.55**	**.02***

Final selected model in bold. **p* <0.5

Supplementary table 4. *Model fitting for retention – skill measure*

		N total obs = 46; N Subjects = 23; N time = 2			Model Fit			LRT Test against comparison	
**Model Specification**	**Model Name**	**Compared Model**	**Model description**	**Random Effects**	**AIC**	**BIC**	**LL**	χ^2	*p*
RE only	Null		Skill ~ (1| Participant)	Participant	49.69	55.18	-21.85		
	SR 1	Null	Skill ~ (1 |Fitness) + (1| Participant)	Participant, fitness	51.69	59.01	-21.85	<.001	>.99
	SR 2	SR 1	Skill ~ 1 | Participant) + (1 | Age)	Participant, age	51.69	59.00	-21.85	.001	.97
FE main effects	SR 4	SR 2	Skill ~ Group + Time + (1 | Participant)	Participant	44.02	53.17	-17.01	9.67	.01*
	SR 5	SR 4	Skill ~ Group + Time + Age + Fitness + Sex + (1 | Participant)	Participant	40.97	55.60	-12.47	9.05	.03*
FE Interactions	**SR 6**		**Skill ~ Group × Time + Age + Fitness + Sex + (1 | Participant)**	**Participant**	**37.21**	**53.67**	**-9.60**		
	SR 8	SR 6	Skill ~ Group × Time × Age + Sex + Fitness + (1 | Participant)	Participant	39.12	61.07	-7.56	4.09	.25
	SR 9	SR 6	Skill ~ Group × Time × Fitness + Sex + Age + (1 | Participant)	Participant	40.82	67.77	-8.41	2.39	.50
	SR 10	SR 6	Skill ~ Group × Time × Sex + Sex + Age + (1 | Participant)	Participant	39.22	61.17	-7.61	3.98	.26

Final selected model in bold. *p <0.5

Supplementary table 5. *Model fitting for retention – force error*

		N total obs = 46; N Subjects = 23; N time = 2			Model Fit			LRT Test against comparison	
**Model Specification**	**Model Name**	**Compared Model**	**Model description**	**Random Effects**	**AIC**	**BIC**	**LL**	χ^2	*p*
RE only	Null		Force Error ~ (1| Participant)	Participant	-126.91	-121.43	66.46		
	ER 1	Null	Force Error ~ (1 |Fitness) + (1| Participant)	Participant, fitness	model failed to converge				
	ER 2	ER 1	Skill ~ 1 | Participant) + (1 | Age)	Participant, age	-125.06	-117.75	66.53	0.15	.70
FE main effects	ER 3	Null	Force Error ~ Group + Time + (1 | Participant)	Participant	-126.97	-117.83	68.49	4.06	.13
	ER 4	Null	Force Error ~ Group + Time + Age + Fitness + Sex + (1 | Participant)	Participant	-128.53	-113.90	72.26	11.61	.04*
**FE Interactions**	**ER 5**		**Force Error ~ Group × Time + Age + Fitness + Sex + (1 | Participant)**	**Participant**	**-130.99**	**-114.53**	**74.49**		
	ER 6	ER 5	Force Error ~ Group × Time × Age + Sex + Fitness + (1 | Participant)	Participant	-131.21	-109.26	77.60	6.22	.10
	ER 7	ER 5	Force Error ~ Group × Time × Fitness + Sex + Age + (1 | Participant)	Participant	-127.82	-105.88	75.91	2.83	.42
	ER 8	ER 5	Force Error ~ Group × Time × Sex + Sex + Age + (1 | Participant)	Participant	-125.83	-103.89	74.92	0.84	.84

Final selected model in bold. *p <0.5

Supplementary table 6. *Model fitting for retention – trial time*

		N total obs = 46; N Subjects = 23; N time = 2			Model Fit			LRT Test against comparison	
**Model Specification**	**Model Name**	**Compared Model**	**Model description**	**Random Effects**	**AIC**	**BIC**	**LL**	χ^2	*p*
RE only	Null		Trial Time ~ (1| Participant)	Participant	61.17	66.65	-27.58		
	TR 1	Null	Trial Time ~ (1 |Fitness) + (1| Participant)	Participant, fitness	63.17	70.48	-27.58	<.001	>.99
	TR 2	Null	Trial Time ~ 1 | Participant) + (1 | Age)	Participant, age	Model failed to converge				
FE main effects	TR 3	Null	Trial Time ~ Group + Time + (1 | Participant)	Participant	61.86	71.01	-25.93	3.30	.19
	TR 4	Null	Trial Time ~ Group + Time + Age + Fitness + Sex + (1 | Participant)	Participant	66.61	81.23	-25.30	4.56	.47
**FE Interactions**	**TR 5**		**Trial Time ~ Group × Time + (1 | Participant)**	**Participant**	**63.36**	**74.34**	**-25.68**		

Final selected model in bold. **p* <0.5

Supplementary Results

Supplementary table 7

Results of linear mixed models for acquisition across skill, force error and trial time measures

Parameter	Estimate (*SE*)	95% CI [lower, upper]	Test *(df)*	*p*
		**Skill**		
Group (reference = rest)	-6.58 (2.75)	-12.01, -1.15	*F*(1, 17.00) = 5.71	**.03***
Block	-0.05 (0.19)	-0.42, 0.32	*F*(1, 19.00) = 2.30	.14
Age	-0.10 (0.03)	-0.17, -0.03	*F*(1, 19.00) = 5.74	**.03***
Younger-old vs older	0.71 (0.27)	0.13, 1.28	*t*(17.06) = 2.59	**.02***
Fitness (VO_2peak)	-0.03 (0.01)	-0.05, -0.01	*F*(1, 17.00) = 7.71	**.01***
Sex (reference = male)	-0.52(0.23)	-0.97, -0.07	*F*(1, 17.00) = 5.18	**.04***
Group × Block	0.44 (0.22)	0.00, 0.89	*F*(1, 19.00)= 3.91	.06
Group × Age	0.10 (0.04)	0.02, 0.18	*F*(1, 17.00) = 5.60	**.03***
Block × Age	0.002 (0.003)	0.00, 0.01	*F*(1, 19.00) = 1.18	.29
Group × Block × Age	-.007 (0.003)	-0.01, 0.00	*F*(1, 19.00) = 4.61	**.05***
Condition × Block trend				
Younger-old (rest)	0.06 (0.02)	0.01, 0.11	*t*(19.00) = 2.66	**.03***
Younger-old (exercise)	0.06 (0.01)	0.03, 0.09	*t*(19.00) = 4.51	**.001***
Older (rest)	0.08 (0.02)	0.03, 0.12	*t*(19.00) = 4.40	**.001***
Older (exercise)	-0.001 (0.02)	-0.04, -0.08	*t*(19.00) = -0.08	>.99
		**Force Error**		
Group (reference = rest)	1.04 (0.47)	0.10, 1.97	*F*(1, 19.76) = 4.81	**.04***
Block	0.004 (0.02)	-0.04, 0.03	*F*(1, 197.96) = 6.57	**.01***
Age	0.01 (0.01)	0.00, 0.02	*F*(1, 19.55) = 2.35	.14
Fitness (VO_2peak)	0.003 (0.002)	0.00, 0.01	*F*(1, 17.00) = 2.71	.12
Sex (reference = male)	0.08 (0.04)	0.00, 0.15	*F*(1, 17.00) = 4.03	.06
Group × Block	-0.05 (0.01)	-0.10, -0.01	*F*(1, 195.00) =2.21	**.03***
Group × Age	-0.02 (0.01)	-0.03, 0.00	*F*(1, 19.78) = 4.82	**.04***
Block × Age	0.001 (<.001)	-0.001, 0.001	*F*(1, 195.00) = 4.93	**.03***
Group × Block × Age	0.001 (<.001)	0.00, 0.001	*F*(1, 195.00) = 5.70	**.02***
Condition × Block trend				
Younger-old (rest)	-0.01 (0.002)	-0.01, -0.001	*t*(112.8) = -2.54	**.02***
Younger-old (exercise)	-0.01 (0.002)	-0.01, -0.003	*t*(35.4) = -4.34	**<.001***
Older (rest)	-0.01 (0.002)	-0.01, -0.002	*t*(72.2) = -3.20	**.004***
Older (exercise)	0.002 (0.002)	-0.002, 0.01	*t*(80.5) = 1.22	.45
		**Trial Time**		
Group (reference = rest)	-2.62 (3.06)	-8.66, 3.42	*F*(1, 19.00) = 0.73	.40
Block	0.83 (0.35)	0.15, 1.51	*F*(1, 19.00) = 6.28	**.02***
Age	0.02 (0.04)	-0.05, 0.09	*F*(1, 19.00) = 3.25	.09
Group × Block	-0.62 (0.41)	-1.44, 0.20	*F*(1, 19.00) = 2.23	.15
Group × Age	.0432 (0.05)	-0.05, 0.13	*F*(1, 19.00) = 0.90	.35
Block × Age	-0.01 (0.01)	-0.02, 0.00	*F*(1, 19.00) = 7.62	**.01***
Younger-old	-0.002 (0.02)	-0.06, 0.05	*t*(19.00) = -0.09	>.99
Older	-0.09 (0.02)	-0.14, -0.04	*t*(19.00) = -4.38	**.001***
Group × Block × Age	0.01 (0.01)	0.00, 0.02	*F*(1, 19.00) = 2.18	.16

Df = degrees of freedom. *F* statistic derived from type III ANOVA. Pairwise comparisons and trends are indented.

**p* >.05

Supplementary table 8

Results of linear mixed models for retention across skill, force error and trial time measures

Parameter	Estimate (*SE*)	95% CI	Test *(df)*	*p*
		**Skill**		
Group (reference = rest)	-0.42 (1.92)	2.05, 9.82	*F*(1, 18) = 1.79	.20
Time (reference = T1)	-0.23 (0.06)	-0.34, -0.12	*F*(1,21) = 10.53	.004*
Age	-0.05 (0.02)	-0.10, 0.00	*F*(1,18) = 4.16	.06
Fitness (VO_2peak)	-0.02 (0.01)	-0.05, 0.00	*F*(1,18) = 2.77	.11
Sex (reference = male)	-0.52(0.23)	-0.97, -0.07	*F*(1,18) = 4.79	**.04***
Group × Time	0.20 (0.08)	0.03, 0.36	*F*(1,21) = 5.98	**.02***
Rest (T2 vs T1)	-0.23 (0.06)	-0.35, -0.11	*t*(21) = -4.11	**.002***
Exercise (T2 vs T1)	-0.03 (0.06)	-0.15, 0.09	*t*(21) = -0.55	>.99
		**Force Error**		
Group (reference = rest)	0.04 (0.04)	-0.04, 0.12	*F*(1, 18) = 0.45	.51
Time (reference = T1)	0.03 (0.01)	0.01, 0.04	*F*(1,21) = 3.92	.06
Age	0.01 (<0.001)	0.00, 0.02	*F*(1,18) = 3.13	.09
Fitness (VO_2peak)	<0.001 (<0.001)	0.00, 0.01	*F*(1,18) = 2.06	.17
Sex (reference = male)	0.09 (0.04)	0.00, 0.18	*F*(1,18) = 4.07	.06
Group × Time	-0.03 (0.01)	-0.05, 0.00	*F*(1,21) = 4.50	**.05***
Rest (T2 vs T1)	0.03 (0.01)	0.01, 0.04	*t*(21) = 2.97	**.01***
Exercise (T2 vs T1)	-0.001 (0.01)	-0.02, 0.02	*t*(21) = -0.10	>.99
		**Trial Time**		
Group (reference = rest)	0.15 0.32	-0.51, 0.80	*F*(1, 18) = 0.35	.56
Time (reference = T1)	0.06 0.08	-0.10, 0.23	*F*(1,21) = 3.02	.10
Group × Time	0.08 (0.12)	-0.16, 0.32	*F*(1,21) = 0.46	.51

Df = degrees of freedom. *F* statistic derived from type III ANOVA.
Pairwise comparisons and trends are indented.
*p >.05
