# Supplementary material for: High-intensity acute exercise impacts motor learning in healthy older adults
Source: NPJ Sci Learn. 2024 Feb 17;9:9. doi: 10.1038/s41539-024-00220-2 (PMC10874400; doi:10.1038/s41539-024-00220-2)
Supplement: Supplementary file 2 — Reporting summary [file 41539_2024_220_MOESM2_ESM.pdf]

nature portfolio

Corresponding author(s): James P Coxon

Last updated by author(s): YYYY-MM-DD

Reporting Summary

Nature Portfolio wishes to improve the reproducibility of the work that we publish. This form provides structure for consistency and transparency in reporting. For further information on Nature Portfolio policies, see our Editorial Policies and the Editorial Policy Checklist.

Statistics

For all statistical analyses, confirm that the following items are present in the figure legend, table legend, main text, or Methods section.

n/a	Confirmed	
☐	☒	The exact sample size (*n*) for each experimental group/condition, given as a discrete number and unit of measurement
☐	☒	A statement on whether measurements were taken from distinct samples or whether the same sample was measured repeatedly
☐	☒	The statistical test(s) used AND whether they are one- or two-sided<br>*Only common tests should be described solely by name; describe more complex techniques in the Methods section.*
☐	☒	A description of all covariates tested
☐	☒	A description of any assumptions or corrections, such as tests of normality and adjustment for multiple comparisons
☐	☒	A full description of the statistical parameters including central tendency (e.g. means) or other basic estimates (e.g. regression coefficient) AND variation (e.g. standard deviation) or associated estimates of uncertainty (e.g. confidence intervals)
☐	☒	For null hypothesis testing, the test statistic (e.g. *F*, *t*, *r*) with confidence intervals, effect sizes, degrees of freedom and *P* value noted<br>*Give P values as exact values whenever suitable.*
☒	☐	For Bayesian analysis, information on the choice of priors and Markov chain Monte Carlo settings
☐	☒	For hierarchical and complex designs, identification of the appropriate level for tests and full reporting of outcomes
☐	☒	Estimates of effect sizes (e.g. Cohen's *d*, Pearson's *r*), indicating how they were calculated

Our web collection on statistics for biologists contains articles on many of the points above.

Software and code

Policy information about availability of computer code

Data collection	LabVIEW (Version)<br>Matlab (Version)<br>Wattbike Hub (app)<br>Polar Beat (app)<br>LabChart 7<br>Powerlab 16/35
Data analysis	RStudio(version 2022.07.2)<br>R packages lme4, LmerTest, mixedpower

For manuscripts utilizing custom algorithms or software that are central to the research but not yet described in published literature, software must be made available to editors and reviewers. We strongly encourage code deposition in a community repository (e.g. GitHub). See the Nature Portfolio guidelines for submitting code & software for further information.

Data

Policy information about availability of data

All manuscripts must include a data availability statement. This statement should provide the following information, where applicable:

- Accession codes, unique identifiers, or web links for publicly available datasets
- A description of any restrictions on data availability
- For clinical datasets or third party data, please ensure that the statement adheres to our policy

Data is available upon request

Research involving human participants, their data, or biological material

Policy information about studies with human participants or human data. See also policy information about sex, gender (identity/presentation), and sexual orientation and race, ethnicity and racism.

Reporting on sex and gender	Sex was included as a fixed factor in the data analysis model. Sex was not included as an interaction effect in the model as this did not improve overall model fit.
Reporting on race, ethnicity, or other socially relevant groupings	Race and ethnicity were not included as variables in the current study. The sample was recruited from the eastern suburbs of Melbourne, Australia. The sample size was small and predominantly caucasian, therefore meaningful conclusions regarding the impact of race or ethnicity were unlikely to be identified based on the current data.
Population characteristics	Participants included in the final dataset were 23 healthy adults aged 55 -75 years (mean age = 66.68 years, SD = 5.32) with no major medical or psychiatric diagnoses. The sample comprised 15 women and 8 men.
Recruitment	Participants were recruited via local community groups including educational services and fitness groups targeted at older age. Due to its nature as an exercise research study, there was likely a self-selection bias toward individuals with higher cardiorespiratory fitness and a greater level of comfort with physical exercise.
Ethics oversight	Ethical approval was provided by Monash University Human Research Ethics Committee.

Note that full information on the approval of the study protocol must also be provided in the manuscript.

Field-specific reporting

Please select the one below that is the best fit for your research. If you are not sure, read the appropriate sections before making your selection.

☐ Life sciences ☒ Behavioural & social sciences ☐ Ecological, evolutionary & environmental sciences

For a reference copy of the document with all sections, see nature.com/documents/nr-reporting-summary-flat.pdf

Behavioural & social sciences study design

All studies must disclose on these points even when the disclosure is negative.

Study description	This study involved quantitative data and a mixed experimental design.
Research sample	Participants were healthy older adults aged 55 - 75 recruited via targeted advertisements in the local area. Participants were predominantly recruited via advertisements to local educational institutions for older adults and gyms with older-age specific classes. The sample was therefore unlikely to be representative, as it comprised predominantly higher fit and more active older adults.
Sampling strategy	Participants were recruited via convenience sampling. A target sample size of 28 participants was determined based on an effect size of d = 0.96 as reported in Stavrinos & Coxon, (2017) for a Time × Exercise Condition interaction to assess motor learning retention (alpha = .05, power = 80%).
Data collection	Exercise data were collected using Polar Beat app and heart rate monitor, with cycling data collected using Wattbike Hub. Additional measures were taken manually with pen and paper, including heart rate, cadence, power output and percieved exertion. Measures were taken at 1 minute intervals during exercise or 5-minute intervals during active rest.<br>Behavioural data were collected using LabVIEW 2015 and collated using Matlab 2018a.<br>During graded fitness test, data was collected using LabChart, with manual measures taken each minute as described above.<br>The researchers were not blind to group allocation or the hypothesis of the research.
Timing	Data were collected across two time periods, one between 01/07/2019 - 18/12/2019, and another period between 29/06/2022 - 28/11/2022.
Data exclusions	Three participants were excluded prior to data analysis due to performance below a pre-established cutoff on a cognitive screening measure. One additional participant was excluded due to technical problems during testing, which resulted in incomplete data.

Non-participation	44 participants commenced participation in the research study, however due to extended COVID-19 lockdowns and university closures between 2020 and 2022, 20 participants withdrew, were no longer able to participate, or ceased responding and did not complete all aspects of the study.
Randomization	Participants were randomly allocated to an active rest or exercise group via random number generation.

Reporting for specific materials, systems and methods

We require information from authors about some types of materials, experimental systems and methods used in many studies. Here, indicate whether each material, system or method listed is relevant to your study. If you are not sure if a list item applies to your research, read the appropriate section before selecting a response.

Materials & experimental systems

n/a	Involved in the study	
☒	☐	Antibodies
☒	☐	Eukaryotic cell lines
☒	☐	Palaeontology and archaeology
☒	☐	Animals and other organisms
☒	☐	Clinical data
☒	☐	Dual use research of concern
☒	☐	Plants

Methods

n/a	Involved in the study	
☒	☐	ChIP-seq
☒	☐	Flow cytometry
☒	☐	MRI-based neuroimaging

Plants

Seed stocks	N/A
Novel plant genotypes	N/A
Authentication	N/A
